# Correlation analysis in clinical and experimental studies

**DOI:** 10.1590/1677-5449.174118

**Published:** 2018

**Authors:** Hélio Amante Miot

**Affiliations:** 1 Universidade Estadual Paulista – UNESP, Faculdade de Medicina de Botucatu, Departamento de Dermatologia e Radioterapia, Botucatu, SP, Brasil.

 It is common for researchers conducting clinical or biomedical studies to be interested in investigating whether the values of two or more quantitative variables change in conjunction in a given individual or object of study. In other words, whether when the value of one variable increases, the value of another tends to increase/ or, inversely, reduce, progressively. There are many different statistical tests that explore the intensity and direction of this mutual behavior of variables, known as correlation tests. 1 ^,^ 2

 The first step in analyzing correlations between two quantitative variables should be to look at a scatter plot, in order to discern whether there is a gradual variability between the sets of variables, whether this variation is monotonic (predominantly increasing or decreasing), if it follows a proportional tendency (linear), and whether the underlying distribution of the data is normal. 2 ^-^ 4 Different combinations of these premises indicate a need for different techniques for correlation analysis. 

**Figure 1:**
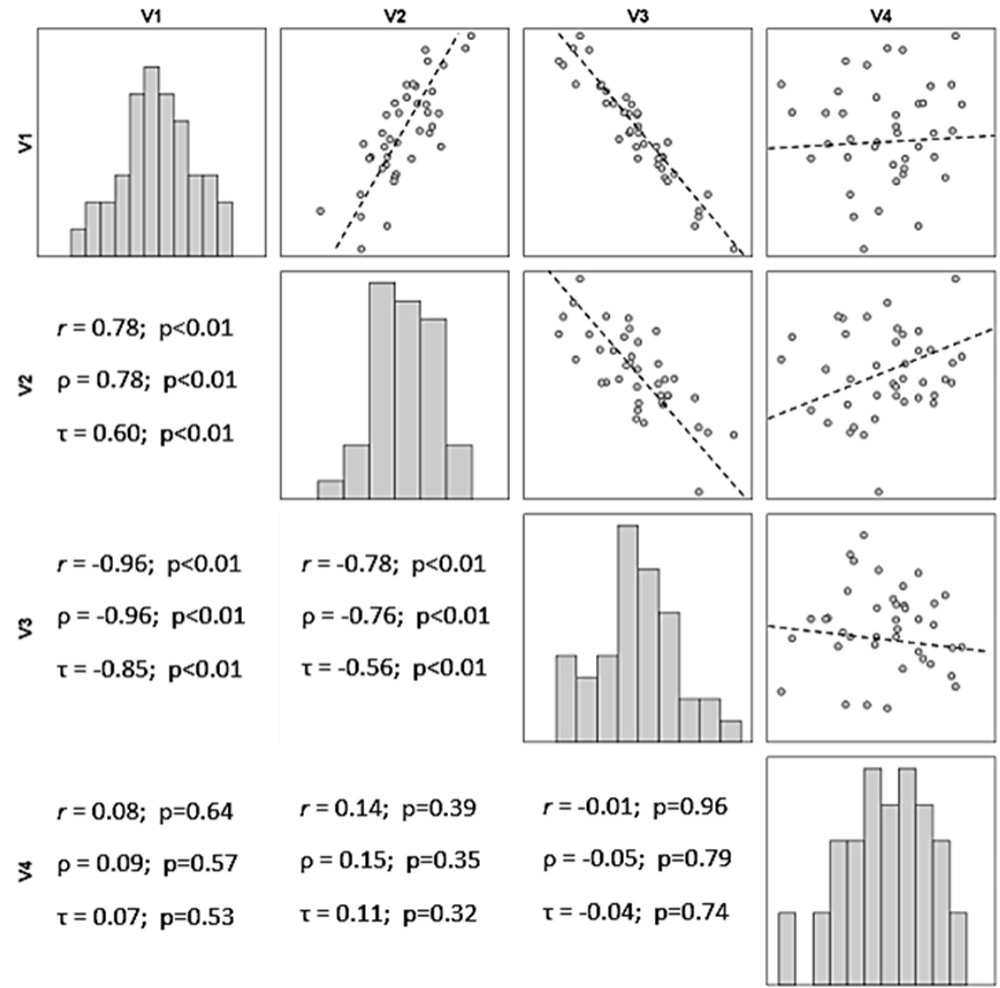
Bar graphs, scatter plots, and correlation coefficients (*r*: Pearson, ρ: Spearman, and τ: Kendall *Tau*-b) for four hypothetical quantitative variables V1, V2, V3, and V4 (n = 40).

Figure 1 illustrates the distribution of values of four hypothetical variables (V1, V2, V3, and V4), which exhibit data that follow a normal distribution (Shapiro-Wilk, p > 0.32). 

 Variables V1 and V2 exhibit simultaneously increasing values, which are distributed around an underlying imaginary (ideal) straight line, which describes the trajectory of the data. It can be stated that there is a positive linear correlation between V1 and V2. For example, Rossi et al. identified a strong positive correlation (ρ = 0.82; p < 0.01) between cores on the Venous Symptoms Clinical Severity Scale and pain in chronic venous disease. 5

 In contrast, variables V1 and V3 exhibit antagonistic behavior: when the values of one increase, the values of the other reduce. It can be stated that there is a negative linear correlation between V1 and V3, just as Ohki and Bellen identified a moderate negative correlation (ρ = -0.65; p < 0.01) between average regional temperature and the incidence of venous thrombosis. 6

 It can also be observed that the values for the correlation between V1 and V3 are closer to the imaginary straight line than the values for the correlation between V1 and V2. This invites the conclusion that the relationship between the values of the variables V1 and V3 is stronger than the relationship between V1 and V2, even though the directions are opposite. 

 Comparisons of the data for V4, whether with V1, V2, or V3, do not reveal gradually increasing or decreasing behavior. This leads to the conclusion that V4 does not exhibit a correlation with the other variables. 

 The most widely-used technique for evaluating the correlation between two quantitative variables is Pearson’s product-moment correlation coefficient, or Pearson’s *r*, which requires that both samples follow a normal distribution and that the relationship between the two variables is linear. 2 ^,^ 7 Failure to adhere to these prerequisites leads to erroneous conclusions, even when working with large sample sizes. 

 However, it is very common that samples of clinical and demographic data do not follow a normal distribution (for example, the distributions of income, quality of life indexes, disease severity indexes, years of study, and number of children). The most widely used options for investigating correlations between variables that do not exhibit normal distributions are the Spearman rank order correlation and the Kendall rank correlation coefficient (*Tau*-b), which substitute the original data for their ordered ranks. 2 ^,^ 7 ^,^ 8 These methods are also used in cases in which at least one of the variables has ordinal characteristics (for example, functional class, educational level, cancer staging, social class). 

 Another advantage of using the Spearman and Kendall nonparametric tests is that they are not restricted to linear correlations, as long as they exhibit monotonic behavior. In other words, they must exhibit a gradual relationship in the same direction (rising or falling) for the whole domain of the data studied. 

**Figure 2:**
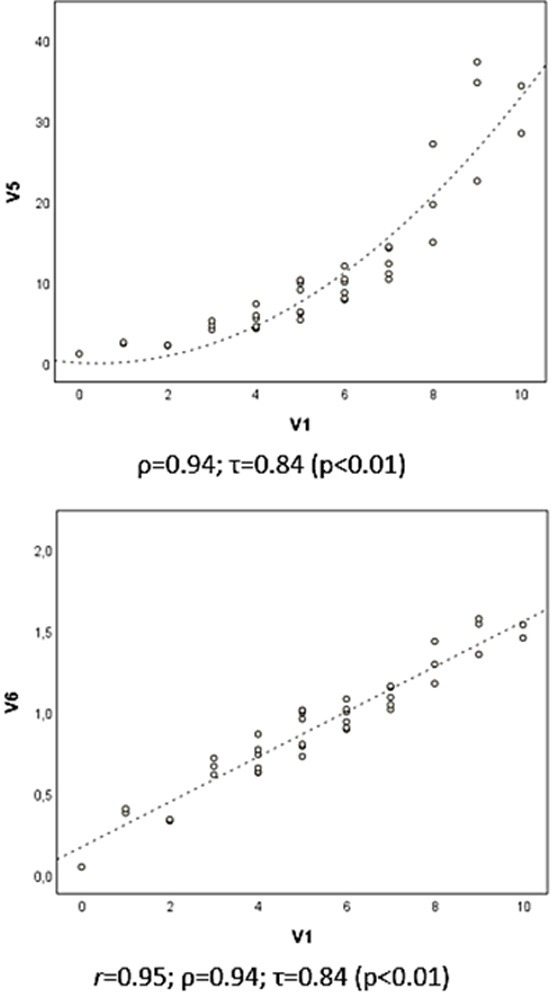
Scatter plots and correlation coefficients for the hypothetical variables V1, V5 and V6 (n = 40), where V6 is the result of log transformation of V5 (V6 = log_10_ V5).

 In Figure 2 , it can be observed that there is no direct proportionality (linear) between the data for V1 and V5; rather there is an increase that is apparently exponential. Since the variation is monotonic (the data for V1 increase as a function of V5), the Spearman and Kendall coefficients can be used to estimate the correlation. In this example, to use the Pearson’s coefficient, it will be necessary to log transform the data to achieve a certain linearity of correlation ( Figure 2 : V1 x V6). It should be noted that the ρ and τ coefficients give the same resultant values for the correlations V1 vs. V5 and V1 vs. V6, since V6 is a transformation of V5 into monotonic data. 

 In biomedical sciences, the Spearman coefficient (ρ or rho) is the most widely used for evaluating the correlation between two quantitative variables, probably because it is similar to the Pearson method, once the data have been substituted for their ordered ranks. However, care should be taken when generalizing conclusions of interpretations of the correlation between the values of the ranks of data from the variables and the original data. 

 In contrast, the Kendall Tau-b coefficient (τ or t_b_), has mathematical properties that make it more robust to extreme data (outliers), give it a greater capacity for populational inference and a smaller estimation error. While significance (p-value) and direction (+ or -) are similar to those of the Spearman method, the coefficient returns less extreme values and interpretation is different, since it signifies the percentage of observed pairs that take the same direction in the sample (agreement) minus the pairs that do not agree. For example, a τ coefficient of 0.60 signifies that 80% of pairs agree, while 20% disagree (τ = 0.80 - 0.20 = 0.60). 9

 Transformation of data (for example, logarithmic, square root, 1/x) in order to obtain a normal distribution to enable Pearson’s coefficient to be tested is a valid option for samples with asymmetrical data distributions ( Figure 2 : V1 x V6). However, it should be borne in mind that, in common with techniques that employ ordered ranks, transformation of data alters the scale between measures and impacts on direct interpretation of the measures of effect. 7

 The magnitude of the effect of the correlation between two or more variables is represented by correlation coefficients, which take values from -1 to +1, passing through zero (absence of correlation). Positive coefficients (*r* > 0) indicate a direct relationship ( Figure 1 : V1 x V2) between variables; while negative coefficients (*r* < 0) indicate an inverse correlation ( Figure 1 : V1 x V3 and V2 x V3). 

 Each correlation test has its own coefficient, demanding its own interpretation. In general, for the coefficients Pearson’s r and Spearman’s ρ, values from 0 to 0.3 (or 0 to -0.3) are biologically negligible; those from 0.31 to 0.5 (or -0.31 to -0.5) are weak; from 0.51 to 0.7 (or -0.51 and -0.7) are moderate; from 0.71 to 0.9 (or -0.71 to 0.9) are strong correlations; and correlations > 0.9 (or < -0.9) are considered very strong. 8

 One peculiarity of Pearson’s *r* coefficient is that the square of its value provides an estimate of the percentage of variability in the values of one variable that is explained by the variability in the other. For example, a coefficient of *r* = 0.7 indicates that 49% of the variability of one variable can be explained by, or is followed by, the variation in the values of the other, in the sample tested. 

 In clinical and biomedical studies, the majority of coefficients with biological significance fall in the range of 0.5 to 0.8 (or -0.5 to -0.8). This is the result of errors of measurement, laboratory techniques, or variation of instruments, which affect the precision of measurements, and also, and primarily, because biological phenomena are affected by multifactorial influences and complex interactions, in which the variation of a single variable cannot totally explain the behavior of another. 2

 Tests of the significance of a correlation between quantitative variables are based on the null hypothesis that there is no correlation between the variables (*r* = 0), which makes the p-value subject to influence both from the dimension of the effect and from the sample size. This means that caution is necessary when interpreting coefficients that result in a weak correlation (r < 0.3), but have highly significant p-values, caused by overly-large sample sizes. Calculation of sample sizes for analysis of correlations has been explored in an earlier edition of this periodical. 10

 Correlation coefficients have inferential properties and, in scientific texts, should preferably be expressed with their 95% confidence intervals and significance (p-value), for example: ρ = 0.76 (95%CI 0.61-0.91), p < 0.01. 11 ^,^ 12 In the case of multiple comparisons, coefficients can be shown on their own, in the form of a matrix, and with their significance indicated to facilitate interpretation of the data, as Brianezi et al. presented the 28 correlations between seven histological parameters in a single table. 13 Special cases involving hundreds or thousands of correlations may demand graphical representation techniques, such as the color heatmaps often used in genome studies, just as Hsu et al. represented 4,930 correlations between (85x58) genomic and metabolomic variables. 14

 In the case of ordinal data with few categories (for example, satisfaction scores, quality of life items, socioeconomic status), investigations based on the polychoric test of correlation may be more robust (smaller type I errors) than using the Spearman and Kendall tests. 15 Although rarely used, there are also methods for assessing the correlations between variables of a categorical nature (for example, Cramér’s V coefficient) and between dichotomous and quantitative variables (for example, the point-biserial correlation coefficient), but these approaches are beyond the scope of this text. 7

 In special situations in which linear correlations between different variables must be analyzed in conjunction (for example, questionnaire items) in order to understand the overall variation in conjunction of the variables, analysis of the correlation between “n” variables can be assessed using the consistency type Intraclass Correlation Coefficient (ICC). There are different ways to analyze ICCs, which result in indicators of different magnitudes. 16 An ICC (random or two-factor mean, mean measures) returns the same value as Cronbach’s α coefficient, used to measure the internal consistency of scales. 17

 The identification of a significant correlation between two or more variables should be interpreted with caution, since statistical analysis does not provide evidence of direct dependence or even of causality between the variables, just that they tend to vary in conjunction. 1 ^,^ 18 ^,^ 19 However, despite the risk of fallacious conclusions of causality or on the basis of results of correlations between variables, correlation tests are important exploratory techniques for investigation of associations between the behavior of groups of variables, facilitating construction of hypothetical models that should then be confirmed by means of dedicated experiments. Indeed, this occurs with ecological clinical studies that often employ correlation techniques for data analysis and provide a basis for subsequent investigations of the phenomena indicated by correlations between indicators and population groups. 20 ^-^ 22 The same applied to genome-wide and proteomic studies, considered exploratory, which study the patterns of correlations of findings with clinical variables in order to indicate models for later confirmation. 19 ^,^ 23

 Indeed, performing multiple correlation tests on a sequence of variables increases the chances of identifying, by chance, correlations described as “spurious”, which should be evaluated in terms of their biological plausibility and confirmed later using appropriate investigation techniques. Use of techniques for correction of p-values adjusted for multiple correlations is always recommended in these conditions. 7 ^,^ 19 ^,^ 24 ^-^ 27

 Another limitation of an inferential nature in correlation analyses is rooted in their incapacity for extrapolation of conclusions to different data intervals or different populations from those studied. 

 Correlation analyses were not developed a priori with the purpose of predicting values or for inference of the participation of multiple variables in the explanation of a phenomenon and there are regression or multivariate analysis techniques that can be used for this purpose. 7 Although there are partial correlation techniques that adjust the correlation values for the behavior of confounding variables (identical to the standardized β coefficient in multivariate linear regression), and polynomial transformation techniques for correction of non-monotonic correlations, an experienced statistics professional should be consulted for planning and execution of analyses of greater complexity. 

 Correlation analyses can also be employed to compare parallelism of measures between two different scales for measurement of the same phenomenon, such as psychometric quality-of-life scales, 28 or clinimetric scales, such as pressure ulcer risk scales. However, researchers very often use them erroneously to test agreement of data or consecutive measures of the same phenomenon (for example, test-retest, 29 calibration of measurement instruments, interrater comparisons), even though there are more appropriate methods for these purposes. 30

 Finally, strategies for evaluation of correlations between variables should be encouraged in clinical and biomedical research, since they maximize understanding of the phenomena studied. However, due to the peculiarities inherent to the different methods, they must be described in detail in the methodology and in the presentation of results. 
