# Supplementary material for: Correlation analysis in clinical and experimental studies
Source: J Vasc Bras. 2018 Oct-Dec;17(4):275–9. doi: 10.1590/1677-5449.174118 (PMC6375260; doi:10.1590/1677-5449.174118)
Supplement: Translation

# Translation: Análise de correlação em estudos clínicos e experimentais

**Authors:** Hélio Amante Miot

**Affiliations:** 1 Universidade Estadual Paulista – UNESP, Faculdade de Medicina de Botucatu, Departamento de Dermatologia e Radioterapia, Botucatu, SP, Brasil.

 É habitual que, em uma pesquisa clínica ou biomédica, seja do interesse do pesquisador investigar se os valores de duas ou mais variáveis quantitativas se modificam de forma conjunta em um mesmo sujeito ou objeto de estudo. Ou seja, quando o valor de uma variável aumenta, o valor de outra tende a aumentar; ou, inversamente, reduza-se progressivamente. Há uma série de testes estatísticos que exploram a intensidade e o sentido desse comportamento mútuo entre variáveis, os chamados testes de correlação 1 ^,^ 2 . 

 O primeiro passo para analisar a correlação entre duas variáveis quantitativas deve ser a visualização do diagrama de dispersão, a fim de identificar se existe uma variabilidade gradual entre os conjuntos de dados, se essa variação é monotônica (predominantemente ascendente ou descendente), se assume uma tendência proporcional (linear) e se a distribuição subjacente dos dados é normal 2 ^-^ 4 . Diferentes combinações dessas premissas indicam diferentes técnicas de análise de correlação. 

 A Figura 1 exemplifica a dispersão dos valores entre quatro variáveis hipotéticas (V1, V2, V3 e V4), as quais apresentam dados aderentes à distribuição normal (Shapiro-Wilk, p > 0,32). 

**Figura 1:** Histogramas, diagramas de dispersão e coeficientes de correlação (*r*: Pearson, ρ: Spearman, e τ: Kendall *Tau* -b) entre quatro variáveis quantitativas hipotéticas V1, V2, V3 e V4 (n = 40).

 As variáveis V1 e V2 apresentam um crescimento simultâneo dos valores, que se distribuem sobre uma linha reta subjacente imaginária (ideal), a qual descreve a trajetória dos dados. Pode-se afirmar que existe correlação linear positiva entre V1 e V2. Da mesma forma, Rossi e colaboradores identificaram forte correlação positiva (ρ = 0,82; p < 0,01) entre os escores da Escala de Gravidade Clínica dos Sintomas Venosos e da escala de dor em doença venosa crônica 5 . 

 As variáveis V1 e V3, em contrapartida, apresentam um comportamento antagônico: enquanto os valores de uma aumentam, os da outra reduzem. Pode-se afirmar que existe correlação linear negativa entre V1 e V3, assim como Ohki e Bellen identificaram moderada correlação negativa (ρ = -0,65; p < 0,01) entre a temperatura regional média e a incidência de trombose venosa 6 . 

 Nota-se ainda que os valores da correlação V1 e V3 aproximam-se mais da linha reta imaginária que os valores da correlação entre V1 e V2. Isso leva à conclusão que a relação entre os valores das variáveis V1 e V3 é mais intensa que a relação entre V1 e V2, apesar dos sentidos serem opostos. 

 As comparações dos dados da V4, sejam com V1, V2 ou V3, não apresentam um comportamento gradualmente ascendente ou descendente. Isso leva à conclusão que V4 não apresenta correlação com as demais variáveis. 

 A técnica mais difundida para a avaliação da correlação entre duas variáveis quantitativas é o coeficiente de correlação produto-momento de Pearson, ou *r* de Pearson, que pressupõe distribuição normal das duas amostras e comportamento linear da relação entre as variáveis 2 ^,^ 7 . A falta de observação dessas premissas leva a conclusões equivocadas, mesmo diante de amostragem numerosa. 

 É, porém, bastante comum que amostras de dados clínicos e demográficos não sigam distribuição normal (por exemplo, distribuição de renda, índices de qualidade de vida, índices de gravidade de uma doença, anos de estudo, número de filhos). As opções mais utilizadas na investigação da correlação entre variáveis que não apresentam distribuição normal são a correlação da ordem de postos de Spearman e o coeficiente de correlação de postos de Kendall (*Tau*-b), que substituem os dados originais por postos ordenados (*ranks*) 2 ^,^ 7 ^,^ 8 . Esses métodos também se impõem em casos que ao menos uma das variáveis apresenta características ordinais (por exemplo, classe funcional, nível de escolaridade, estadiamento de câncer, classe social). 

 Outra vantagem do uso dos testes não paramétricos de Spearman e de Kendall é que eles não se restringem a correlações lineares, desde que apresentem comportamento monotônico. Ou seja, eles devem apresentar uma relação gradual no mesmo sentido (ascendente ou descendente) para todo o domínio de dados estudado. 

 Na Figura 2 , observa-se que não ocorre proporcionalidade direta (linear) entre a variação dos dados de V1 e V5, mas um incremento aparentemente exponencial. Como a variação é monotônica (os dados de V1 oscilam em um crescente em função de V5), os coeficientes de Spearman e Kendall podem estimar a correlação. Já nesse exemplo, para o emprego do coeficiente de Pearson, seria necessária a transformação logarítmica dos dados para garantir certa linearidade da correlação ( Figura 2 : V1 x V6). Destaque-se que os coeficientes ρ e τ resultaram nos mesmos valores para a correlação V1 *versus* V5 e para V1 *versus* V6, já que V6 se tratava de uma transformação de V5 em dados monotônicos. 

**Figura 2:** Diagramas de dispersão e coeficientes de correlação entre as variáveis hipotéticas V1, V5 e V6 (n = 40), sendo V6 a transformação logarítmica de V5 (V6 = log_10_ V5).

 O coeficiente de Spearman (ρ ou rho) é o mais empregado em ciências biomédicas para avaliar a correlação entre duas variáveis quantitativas, provavelmente porque seja semelhante ao método de Pearson, desde que os dados sejam substituídos por postos ordenados (*ranks*). O que, porém, inspira cuidado na generalização das conclusões quanto à interpretação da correlação entre os valores dos *ranks* dos dados das variáveis e não dos dados originais. 

 O coeficiente de Kendall Tau-b (τ ou t_b_), em contrapartida, apresenta propriedades matemáticas que o tornam mais robusto a dados extremos (*outliers* ), de maior capacidade de inferência populacional e menor erro de estimativa. Apesar da significância (p-valor) e o sentido (+ ou -) se aproximarem do método de Spearman, seu coeficiente apresenta valores menos proeminentes e sua interpretação é diferenciada, significando o percentual de pares observados que assumem o mesmo sentido na amostra (concordantes) menos os pares discordantes. Por exemplo, um coeficiente τ de 0,60 representa que 80% dos pares são concordantes, enquanto 20% são discordantes (τ = 0,80 - 0,20 = 0,60) 9 . 

 A transformação dos dados (por exemplo, logaritmo, raiz quadrada, 1/x) a fim de obter sua normalização para testar o coeficiente de Pearson é uma alternativa válida em amostras com distribuição dos dados assimétricas ( Figura 2 : V1 x V6). Entretanto, deve-se ter em mente que, da mesma forma que as técnicas que empregam postos ordenados (*ranks*), as transformações dos dados alteram a escala entre as medidas, influenciando a interpretação direta das medidas de efeito 7 . 

 A magnitude do efeito da correlação entre duas ou mais variáveis é representada pelos coeficientes de correlação, que assumem valores de -1 até +1, passando pelo zero (ausência de correlação). Coeficientes positivos (*r* > 0) indicam correlação direta ( Figura 1 : V1 x V2) entre as variáveis; já coeficientes negativos (*r* < 0) significam uma correlação inversa ( Figura 1 : V1 x V3 e V2 x V3). 

 Cada teste de correlação apresenta um coeficiente individualizado, que demanda uma interpretação própria. De forma geral, para os coeficientes *r* de Pearson e ρ de Spearman, valores entre 0 e 0,3 (ou 0 e-0,3) são biologicamente desprezíveis; entre 0,31 e 0,5 (ou -0,31 e -0,5) são correlações fracas; entre 0,51 e 0,7 (ou -0,51 e -0,7) são moderadas; entre 0,71 e 0,9 (ou -0,71 e 0,9) são correlações fortes; e > 0,9 (ou < -0,9) são consideradas muito fortes 8 . 

 Uma particularidade do coeficiente *r* de Pearson é que o quadrado do seu valor representa uma estimativa do percentual de variabilidade dos valores de uma variável que é explicado pela variabilidade da outra. Por exemplo, um coeficiente *r* = 0,7 significa que 49% da variabilidade de uma variável pode ser explicada, ou é acompanhada pela variação dos valores da outra na amostra testada. 

 Em estudos clínicos e biomédicos, a maior parte dos coeficientes com significado biológico situa-se entre 0,5 e 0,8 (ou -0,5 e -0,8). Isso decorre tanto da existência de erros de mensuração, técnicas laboratoriais ou variação de instrumentos, que interferem na exatidão e na precisão das medidas, mas, principalmente, porque fenômenos biológicos sofrem influências multifatoriais e interações complexas, em que a variação de apenas uma variável não explica totalmente o comportamento de outra 2 . 

 Os testes de significância da correlação entre variáveis quantitativas se baseiam na hipótese nula que não existe nenhuma correlação entre as variáveis (*r* = 0), sujeitando o p-valor à influência tanto da dimensão do efeito quanto do tamanho amostral. Isso exige cautela na interpretação de coeficientes que resultem em baixa correlação (r < 0,3), mas que apresentem p-valores muito significativos, decorrentes de amostras superdimensionadas. O cálculo do tamanho amostral para análise de correlação já foi explorado anteriormente nesta revista 10 . 

 Os coeficientes de correlação apresentam propriedades inferenciais e devem ser preferencialmente expressos, em textos científicos, pelo seu intervalo de confiança de 95% e a significância (p-valor), por exemplo: ρ = 0,76 (IC95% 0,61-0,91), p < 0,01 11 ^,^ 12 . No caso de múltiplas comparações, o coeficiente pode ser apresentado de forma isolada, na forma de uma matriz, e com sua significância sinalizada para facilitar a leitura dos dados, como Brianezi e colaboradores representaram as 28 correlações de sete parâmetros histológicos em uma tabela 13 . Casos especiais que envolvam centenas ou milhares de correlações podem exigir técnicas de representação gráfica, como os mapas térmicos de cor (*heatmap*) frequentemente usados em estudos genômicos, como Hsu e colaboradores representaram 4.930 correlações entre (85x58) variáveis genômicas e metabolômicas 14 . 

 Em casos de dados ordinais com poucas categorias (por exemplo, escores de satisfação, itens de qualidade de vida, nível socioeconômico), a exploração baseada no teste de correlação policórica pode ser mais robusta (menor erro tipo I) que os testes de Spearman e Kendall 15 . Ainda, apesar de pouco utilizadas, há também formas de se avaliar a correlação entre variáveis de natureza categóricas (por exemplo, coeficiente V de Cramér) e entre variáveis dicotômicas e quantitativas (por exemplo, coeficiente de correlação ponto-bisserial), todavia, essas abordagens ultrapassam o escopo deste texto 7 . 

 Em situações especiais em que a correlação linear entre diferentes variáveis deva ser analisada conjuntamente (por exemplo, itens de um questionário) visando a compreensão da variação conjunta global das variáveis, a análise de correlação entre “n” variáveis pode ser avaliada pelo coeficiente de correlação intraclasse (CCI) do tipo consistência. Há diferentes formas de análise do CCI, que levam a indicadores de diferentes magnitudes 16 . O CCI (aleatório ou misto de dois fatores, medidas médias) retorna o mesmo valor do coeficiente α de Cronbach, usado para medir a consistência interna de escalas 17 . 

 A identificação de uma correlação significativa entre duas ou mais variáveis deve ser interpretada com cautela, visto que a análise estatística não fornece evidências de dependência direta ou mesmo de causalidade entre as variáveis, mas apenas que elas tendem a variar conjuntamente 1 ^,^ 18 ^,^ 19 . Entretanto, apesar do risco de falácia ao se concluir causalidade a partir de resultados de correlação entre variáveis, os testes de correlação são importantes técnicas exploratórias para a investigação de associação entre o comportamento de grupos de variáveis, favorecendo a elaboração de modelos hipotéticos que devam ser confirmados posteriormente por meio de experimentos dedicados. Aliás, isso ocorre com estudos clínicos ecológicos, que frequentemente empregam técnicas de correlação na análise de seus dados e servem de base para investigação posterior dos fenômenos indicados pela correlação entre indicadores e grupos populacionais 20 ^-^ 22 . Ou ainda estudos tipo *genome-wide* e de proteômica, ditos exploratórios, que estudam os padrões de correlações dos achados com as variáveis clínicas em busca da indicação de modelos a serem comprovados posteriormente 19 ^,^ 23 . 

 Inclusive, quando se realizam múltiplos testes de correlação em uma sequência de variáveis, aumentam-se as chances de se identificar, ao acaso, correlações ditas “espúrias”, que devem ser avaliadas quanto à plausibilidade biológica e comprovadas posteriormente por técnicas adequadas de investigação. O emprego de técnicas de correção dos p-valores ajustadas para as correlações múltiplas é sempre indicado nessas condições 7 ^,^ 19 ^,^ 24 ^-^ 27 . 

 Outra limitação de natureza inferencial das análises de correlação baseia-se na incapacidade de extrapolação das conclusões para outros intervalos de dados ou para populações diferentes dos estudados. 

 Análises de correlação não foram desenvolvidas *a priori* com a finalidade de predição de valores ou da inferência da participação de múltiplas variáveis na explicação de um fenômeno, havendo técnicas de regressão ou de análise multivariadas com essas finalidades 7 . Apesar de existirem técnicas de correlação parcial que ajustam os valores da correlação para o comportamento de variáveis de confusão (idêntico ao coeficiente β padronizado em regressão linear múltipla), assim como técnicas de transformação polinomial para corrigir correlações não monotônicas, um profissional estatístico experiente deve ser consultado para o planejamento e a execução de análises de maior complexidade. 

 Análises de correlação podem também ser empregadas na comparação do paralelismo das medidas entre duas escalas diferentes para a medida do mesmo fenômeno, como escalas psicométricas de qualidade de vida 28 , ou clinimétricas, como escalas de risco para úlcera por pressão. Contudo, muitas vezes, elas são utilizadas por pesquisadores no sentido equivocado de se testar a concordância dos dados ou medidas consecutivas do mesmo fenômeno (por exemplo, teste-reteste 29 , calibragem de instrumentos de medida, comparação entre juízes), enquanto existem métodos mais adequados para essas finalidades 30 . 

 Finalmente, as estratégias de avaliação da correlação entre variáveis devem ser incentivadas em pesquisa clínica e biomédica, uma vez que maximizam a compreensão do fenômeno estudado. Entretanto, devido às particularidades inerentes aos seus diversos métodos, elas devem ser detalhadamente descritas na metodologia e na apresentação dos resultados.
